# Supplementary material for: FoodScribe: an open-source semantic framework for nutrient estimation from free-text dietary records
Source: medRxiv. 2026 Jul 17:2026.07.15.26358181. Preprint. [Version 1] doi: 10.64898/2026.07.15.26358181 (PMC13409120; doi:10.64898/2026.07.15.26358181)
Supplement: 1 [file NIHPP2026.07.15.26358181V1-supplement-1.pdf]

medRxiv preprint doi: https://doi.org/10.64898/2026.07.15.26358181; this version posted July 17, 2026. The copyright holder for this preprint (which was not certified by peer review) is the author/funder, who has granted medRxiv a license to display the preprint in perpetuity.
It is made available under a CC-BY 4.0 International license .

Supplementary Figures

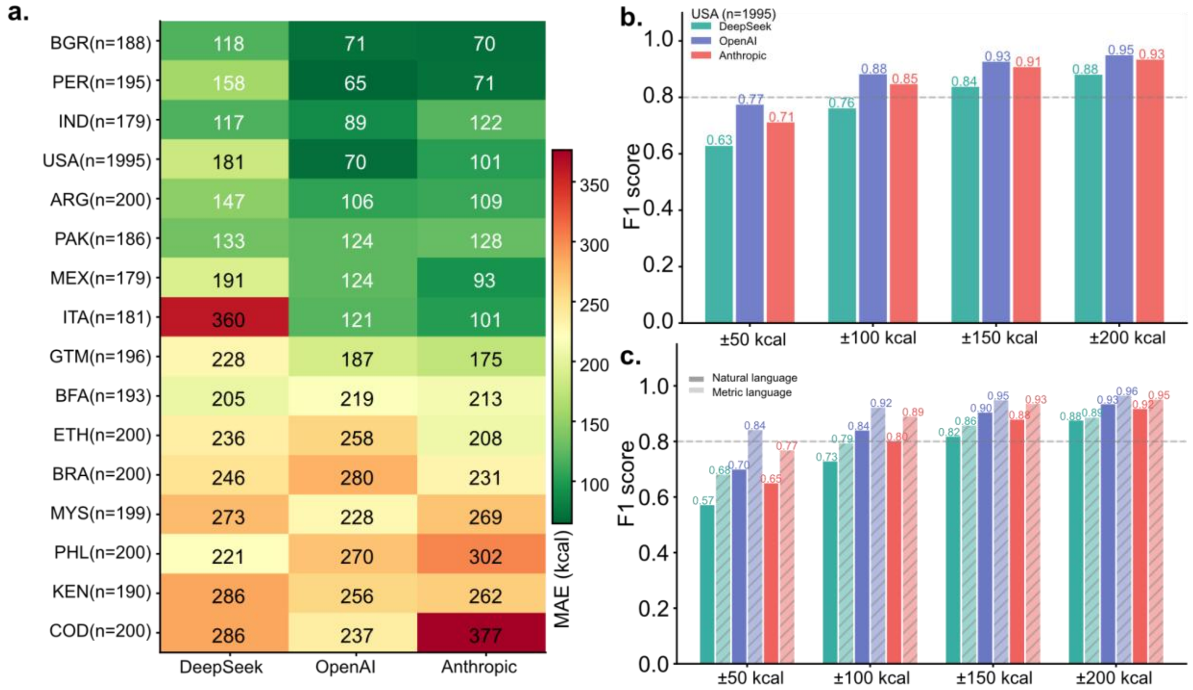

Figure S1: Evaluation of LLM-based nutrient estimation using the Nutribench dataset. (a) Mean absolute error for caloric estimation (kcal) of meal description from 16 countries using three-LLM providers, NutriBench curated ground truth values (n=4,881 meals).Countries are ordered by ascending average MAE across models. Cell color represents MAE magnitude (green = lower, red = higher). Sample sizes per country are indicated in parentheses. (b) Tolerance-based F1 scores for caloric estimation in US-based meals (n=1,995) at four absolute tolerance thresholds (±50, ±100, ±150, and ±200 kcal). A prediction was classified as a true positive if it fell within the specified tolerance of the curated ground truth value; false positives and false negatives represent systematic overestimates and underestimates beyond tolerance, respectively. (c) Tolerance-based F1 scores for caloric estimation in US-based meals stratified by serving description format: natural language (solid bars) versus metric/gram-based descriptions (hatched bars), at the same four tolerance thresholds as in (b). Metric descriptions consistently yielded higher F1 scores across all models and thresholds.

medRxiv preprint doi: https://doi.org/10.64898/2026.07.15.26358181; this version posted July 17, 2026. The copyright holder for this preprint (which was not certified by peer review) is the author/funder, who has granted medRxiv a license to display the preprint in perpetuity.
It is made available under a CC-BY 4.0 International license .

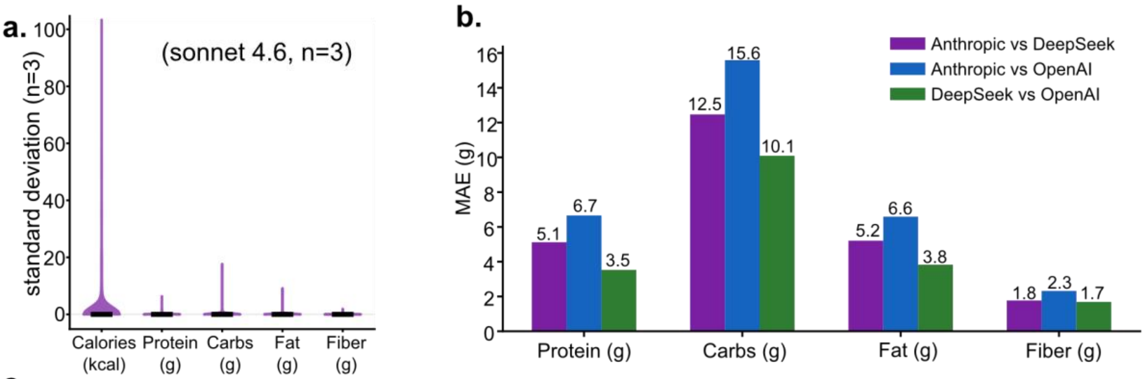

Figure S2: Inter-model agreement in nutrient estimation in MED dietary records. (a) Model reproducibility of macronutrient estimates for claude-sonnet-4.6 across three independent runs (n=3), demonstrates high run-to-run consistency. (b) Pairwise means absolute error (MAE) between LLM providers (Anthropic vs. DeepSeek, purple; Anthropic vs. OpenAI, blue; DeepSeek vs. OpenAI, green), suggesting that defined nutrients are estimated more reliably across models.

medRxiv preprint doi: https://doi.org/10.64898/2026.07.15.26358181; this version posted July 17, 2026. The copyright holder for this preprint (which was not certified by peer review) is the author/funder, who has granted medRxiv a license to display the preprint in perpetuity.
It is made available under a CC-BY 4.0 International license .

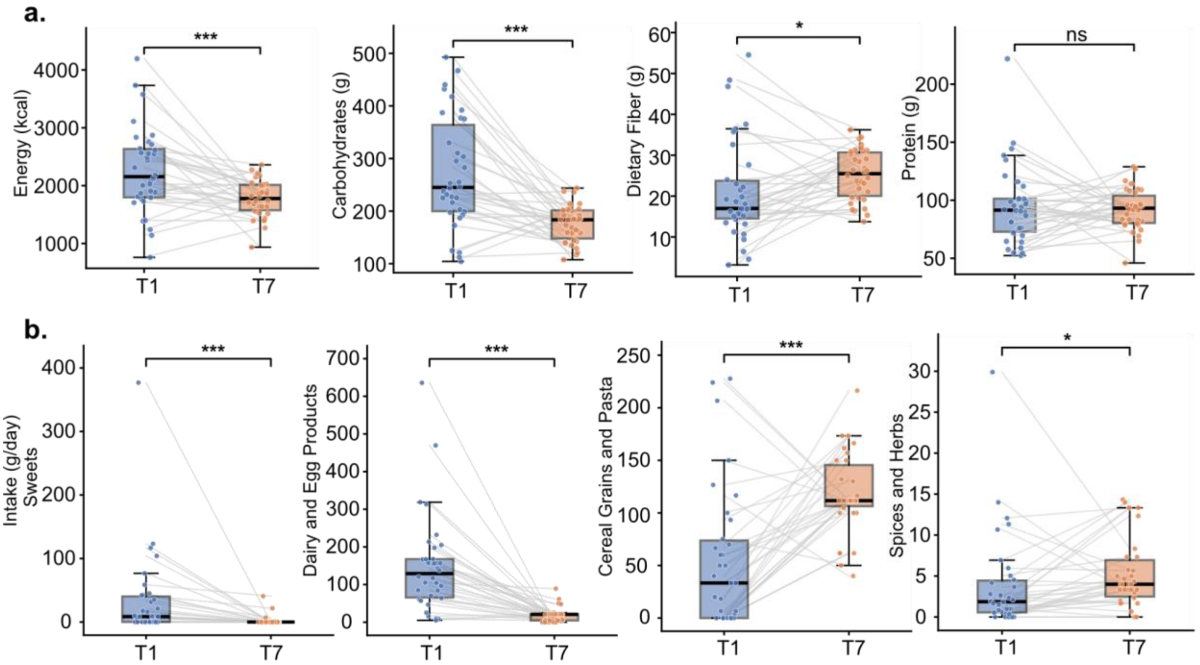

Figure S3: **FoodScribe-derived macronutrient and food group changes between habitual (T1) and Mediterranean diet (T7) phases in a dietary intervention cohort.** (a) Paired boxplots comparing macronutrient intake at T1 (habitual diet, blue) and T7 (Mediterranean diet, orange) across 52 participants. Lines connecting paired observations illustrate individual-level trajectories, highlighting substantial inter-individual variability in the magnitude of dietary change. **(b)** Paired boxplots of food group intake (g/day) between T1 and T7 for selected categories. Statistical significance was assessed by Wilcoxon signed-rank test; *p < 0.05, ***p < 0.001.

medRxiv preprint doi: https://doi.org/10.64898/2026.07.15.26358181; this version posted July 17, 2026. The copyright holder for this preprint (which was not certified by peer review) is the author/funder, who has granted medRxiv a license to display the preprint in perpetuity.
It is made available under a CC-BY 4.0 International license .

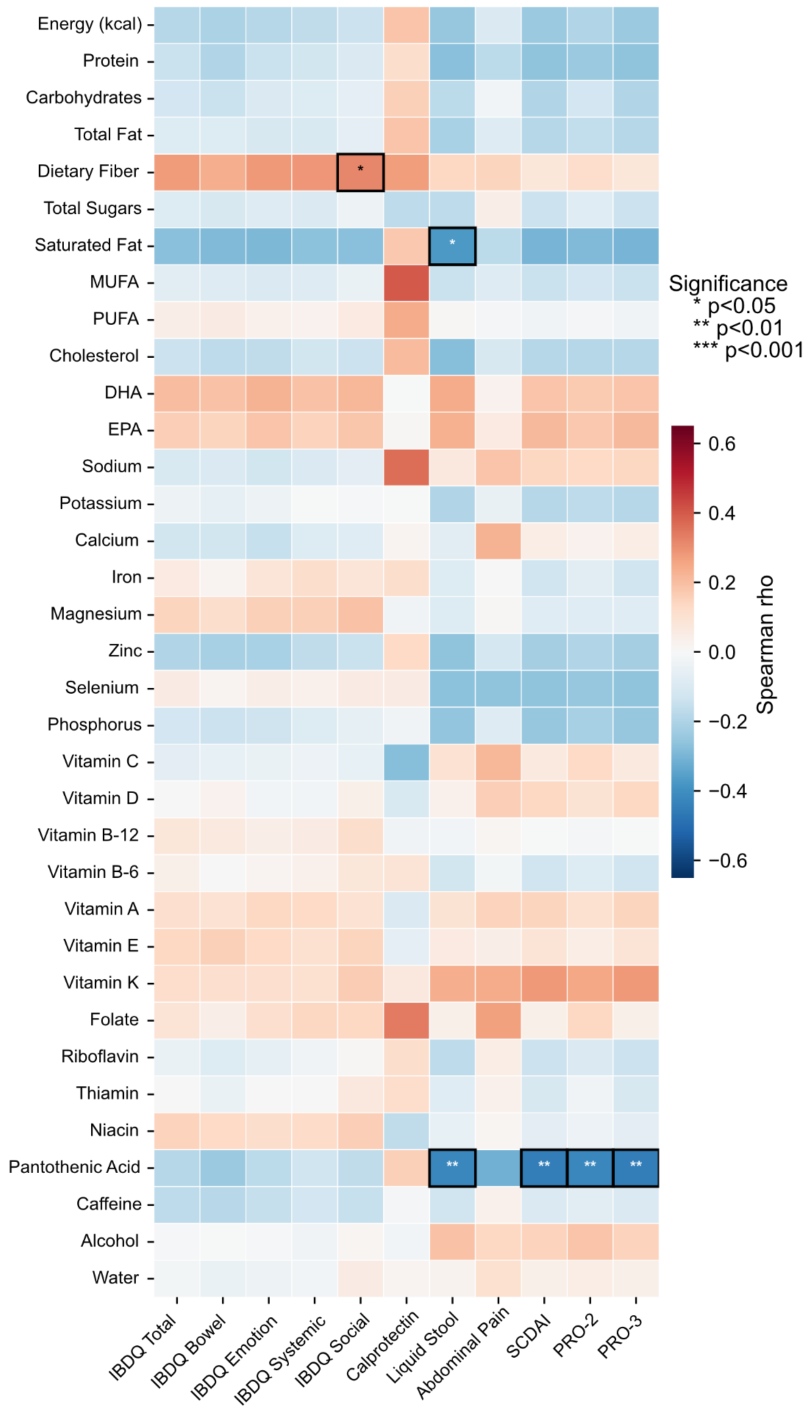

Figure S4: **Spearman Correlation Between Baseline Dietary Intake and Clinical Disease Indices**. Heatmap depicting Spearman correlation coefficients (ρ) between 35 dietary nutrients and 11 clinical indices measured at baseline (V1) in IBD patients and controls. Nutrients (rows) include macronutrients, fatty acids, vitamins, minerals, and other dietary components derived from 3-day averaged dietary records. Clinical indices (columns) include IBDQ subscores (Total, Bowel, Emotion, Systemic, Social), fecal calprotectin, liquid stool frequency, abdominal pain, SCDAI, PRO-2, and PRO-3. Cell color reflects the Spearman ρ value (red = positive correlation, blue = negative correlation; scale clamped at ±0.65). Asterisks denote statistically significant correlations (* p<0.05, ** p<0.01, *** p<0.001); no correction for multiple comparisons was applied.

medRxiv preprint doi: https://doi.org/10.64898/2026.07.15.26358181; this version posted July 17, 2026. The copyright holder for this preprint (which was not certified by peer review) is the author/funder, who has granted medRxiv a license to display the preprint in perpetuity.
It is made available under a CC-BY 4.0 International license .

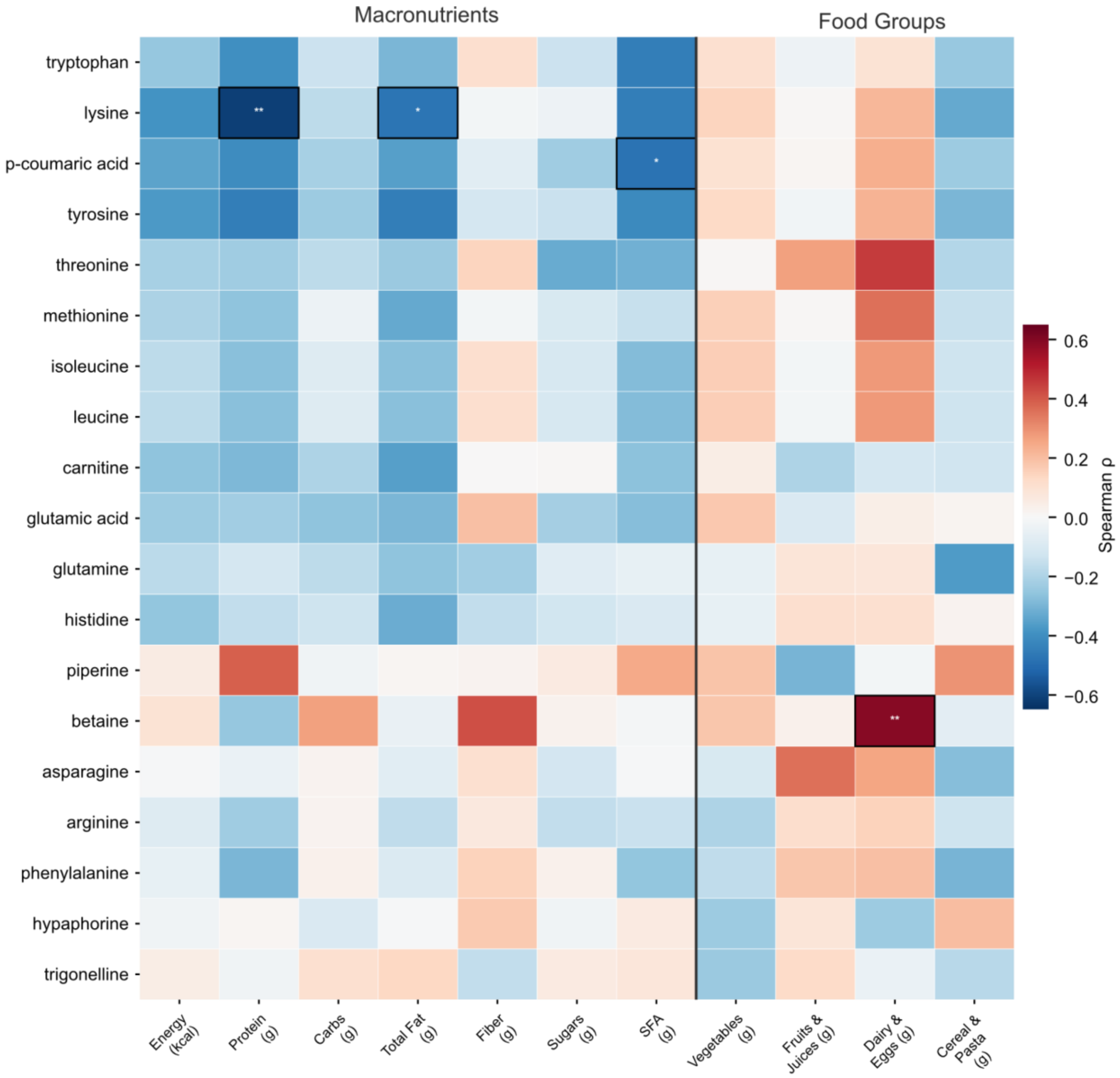

Figure S5: Association Between Plasma Metabolites and Dietary Intake. Heatmap displaying Spearman correlation coefficients (ρ) between 19 plasma metabolites measured at follow-up (V2) and dietary intake at the corresponding timepoint (T7) in n=19 subjects. Plasma metabolite levels (rows) were derived from targeted mass spectrometry and represent mean peak area across technical replicates. Cell color reflects the Spearman ρ (red = positive, blue = negative; scale clamped at ±0.65). Cells with $p<0.05$ are outlined in black and annotated with asterisks (* $p<0.05$, ** $p<0.01$, *** $p<0.001$); no correction for multiple comparisons was applied.
